# Arsenic trioxide treatment of rabbit liver VX-2 carcinoma via hepatic arterial cannulation-induced apoptosis and decreased levels of survivin in the tumor tissue

**DOI:** 10.3325/cmj.2013.54.12

**Published:** 2013-02

**Authors:** Hong Li, Jian Gong, Xuyuan Jiang, Haibo Shao

**Affiliations:** 1 Department of Radiology, First Affiliated Hospital of China Medical University, Shenyang, P. R. China; 2 Department of Clinical Pharmacy, School of Life Science and Biopharmaceutics, Shenyang Pharmaceutical University, Shenyang, P. R. China; The first two authors contributed equally.

## Abstract

**Aim:**

To investigate the role of tumor apoptosis-inhibitory protein survivin in arsenic trioxide-induced apoptosis in VX-2 carcinoma in the rabbit liver by means of transcatheter arterial chemoembolization.

**Methods:**

Sixteen rabbits with 32 implanted hepatic VX-2 tumors were randomly divided into two groups. The experimental group received 2 mg of arsenic trioxide and 1 mL of ultra-fluid lipiodol co-injected via hepatic arterial cannulation and the control group received only 1 mL of lipiodol. Animals were sacrificed 3 weeks after trans-catheterial arterial chemoembolization. Tumor tissue and tumor-peripheral tissue were collected for analysis. Terminal deoxynucleotidyl transferase-mediated dUTP nick-end-labeling staining was used to assess tumor cells apoptosis. Immunohistochemistry was used to assess the presence of survivin protein. Reverse transcription polymerase chain reaction was used to determine the expression of survivin gene.

**Results:**

The number of apoptotic cells significantly increased in the tumor tissue (5.20 ± 0.60%) compared to tumor-peripheral tissue (1.29 ± 0.42%) of the arsenic trioxide-treated group. Survivin expression levels in the tumor tissue were significantly reduced in arsenic trioxide-treated group (7.68 ± 0.65) compared to the control group (35.30 ± 4.63).

**Conclusion:**

Transcatheter arterial chemoembolization with arsenic trioxide induced apoptosis of VX-2 carcinoma, in which tumor apoptosis-inhibitory protein survivin may have played a role.

Hepatocellular carcinoma is the most common type of malignancy in Asia (1). Treatment of patients with unresectable hepatocellular carcinoma is conducted with transcatheter arterial chemoembolization (TACE) (2). Inhibition of apoptosis plays an important role in the generation of malignant tumors, as well as their development and metastasis. Apoptosis is a physiological process important for the preservation of homeostasis and morphogenesis of tissues (3). Many chemotherapeutical drugs treat malignant tumors by interfering with pathological apoptosis regulation of tumor cells. Inducing apoptosis in tumor cells is also the operational principle of arsenic trioxide (ATO), an anticancer drug used in traditional medicine for many centuries (3,4).

Survivin has recently been identified as an inhibitor of apoptosis protein (IAP) (5) with still unclear pathophysiological functioning. Survivin has a unique structure and is expressed in numerous human cancers and during embryo development (6,7), but not in the adult differentiated tissues (8). Thus, survivin may serve as a new target for diagnosis and treatment of malignant tumors (9). The present study investigated the involvement of survivin in ATO-induced apoptosis.

## Methods

### Tumor implantation in rabbit livers

VX-2 cell line originated from a papilloma transformed by the Shope papilloma virus. Active tumor tissues, obtained from rabbits inoculated with VX-2 tumors (VX-2 squamous carcinoma cell line), were implanted into the rabbit livers.

The tumor tissue was first washed with 0.9% NaCl solution, and divided into 1 mm pieces for implantation. The rabbits were anesthetized with intramuscular injection of sodium pentobarbital (30 mg/kg) and immobilized on a surgical table for stereotaxy. Following skin disinfection, a median incision was made below the xiphoid to expose the liver. A 1-2 mm deep cut was made in liver tissue with an ophthalmologic forceps and one prepared tumor tissue piece was implanted. Two tumors were implanted in each rabbit. A single dose of intramuscular penicillin was used to prevent infection. Experiments were carried out 3 weeks after tumor implantation, which is a period required for tumor cells growth.

### Experimental procedure and tissue sampling

Sixteen Japanese white rabbits (2000-2500 g; male:female = 1:1) were randomly divided into two groups using a table of random numbers (16 tumors each group). In the experimental group, 1 mL of ultra-fluid lipiodol (UFLP) with 2 mg ATO (Sigma Chemical Co., St. Louis, MO, USA) was injected into the hepatic artery, while the control group received 1 mL UFLP.

Animals were sacrificed by an intravenous dose of sodium pentobarbital three weeks after the transcatheter arterial chemoembolization. Tumor tissue and tumor peripheral tissue within 2 cm of the tumor margin was collected. A part of the excised tissues was used for terminal deoxynucleotidyl transferase-mediated dUTP nick-end-labeling (TUNEL) staining and immunohistochemical analysis, and the rest was used for reverse transcription polymerase chain reaction (RT-PCR). All experiments and surgical procedures were approved by the Institutional Animal Care and Use Committee at China Medical University, which complied with the National Institute of Health Guide for the Care and Use of Laboratory Animals, and all efforts were made to minimize animal suffering

### Detection of apoptotic cells

To investigate whether ATO-induced apoptosis, the distribution and number of TUNEL-positive cells were compared between the experimental and control group. The excised tissues were fixed in 10% formalin for 24 hours, then embedded in paraffin, and sectioned into 3-μm thick sections using a sliding microtome. The slices were subjected to hematoxylin and eosin (HE) and TUNEL staining. For HE staining, the slices were stained with alum hematoxylin for 4-minute to visualize the nuclei, then washed with 0.3% acid alcohol and stained with eosin for 2 minutes. DNA fragmentation was examined in tissue sections applying a modified TUNEL method (10) using an in situ Apoptosis Detection Kit (ApopTag; Oncor, Gaithersburg, MD, USA). In brief, multiple fragmented 3′-OH ends were labeled with digoxigenin-dUTP in the presence of terminal deoxynucleotidyl transferase. The slices were then counterstained with methylgreen. TUNEL-positive cells in the liver slices were counted using a conventional light microscope under a magnification of 400. To determine the average frequencies of apoptotic cancer cells, 5 slides of each sample and 20 microscopic fields were randomly selected and counted, and examined by three observers. The results were expressed as apoptotic index (AI), which was determined by the following formula: number of apoptotic cells/number of the cells observed ×100%. Brown nucleus was assessed as a positive apoptotic cell.

### Immunohistochemistry

To assess changes in the survivin levels, we used immunohistochemistry.. The slices were deparaffinized in xylene and rehydrated with gradual ethanol to water. To expose antigen epitopes, the slices were heated in a sodium citrate buffer (pH 6.0) in a microwave at a power of 750W for 3.5 minutes. Treated slices were stored at room temperature for 30 minutes and washed in phosphate-buffered saline (PBS, pH 7.4). After preincubation in methanol with 3% H_2_O_2_ for 20 minutes, slices were blocked by serum-free blocking solution at room temperature for 10 minutes (Dako, Carpinteria, CA, USA) and incubated with anti-survivin monoclonal antibody (ab-1 clone 8 E2, Neomarkers, Westinghouse, CA, USA) at a 1:200 dilution at 4°C overnight. After washing in PBS, the slices were incubated with biotinylated rabbit anti-mouse immunoglobulin (Dako-patts, Glostrup, Denmark) at a 1:200 dilution for 30 minutes, followed by peroxidase-conjugated streptavidine (Dako-patts) at a 1:300 dilution for 30 minutes. Finally, they were subjected to 3, 30-diaminobenzidine and counterstained by hematoxylin.

### Gene expression analysis by RT-PCR

To further confirm survivin expression in rabbit liver tumor, we verified protein expression with complementary techniques. The homogenate of the tumor tissue and tumor peripheral tissue were both used for RT-PCR. Total RNA was isolated using Trizol method according to the manufacturer’s instructions (Invitrogen, Carlsbad, CA, USA). The sequence of the forward primer for survivin was 5′-GAC CAC CGC ATC TCT ACA TTC AAG A-3′ and of the reverse primer 5′-TGA AGC AGA AGA AAC ACT GGG C-3′; and the sequence of the forward primer for glyceraldehyde-3-phosphate dehydrogenase (GAPDH) was 5′-TTA GCA CCC CTG GCC AAG G-3′ and of the reverse primer 5′-CTT ACT CCT TGG AGG CCA TG-3′. GAPDH was used as the internal reference. RT-PCR was performed according to the manufacturer’s protocol (Invitrogen). Reaction products were separated on a 2% agarose gel with survivin detected at 139 bp and GAPDH at 200 bp. Gels were photographed and integrated density values (IDV) were calculated by a Chemi Imager 5500 V2.03 software (AlPha InnCh, Miami, FL, USA).

### Statistical analysis

All the experiments were repeated in triplicate and the data were presented as mean ± standard deviation (SD). Statistical analysis was condcuted with SPSS software, version 12.0 (SPSS Inc., Chicago, IL, USA). The analysts were blind to the treatment of each group. *P* < 0.05 was considered statistically significant. To verify consistent mRNA loading among the gels, some blots were probed for GAPDH and the ratio with survivin was determined. There was no significant difference between the density percent control and the ratio with GAPDH; therefore, the analyses for the percent control are presented for each blot. The comparisons of the apoptotic index (AI) and survivin expression between tumor-peripheral and tumor tissues in experimental and control groups were made using one-way ANOVA. When the F-value indicated significance, least-significant difference (LSD) or Tamhane’s T2 post hoc comparisons were made as appropriate to correct for multiple comparisons. All *P*-values were two-tailed.

## Results

### Apoptosis of VX-2 carcinoma cells induced by ATO

**Figure 1:**
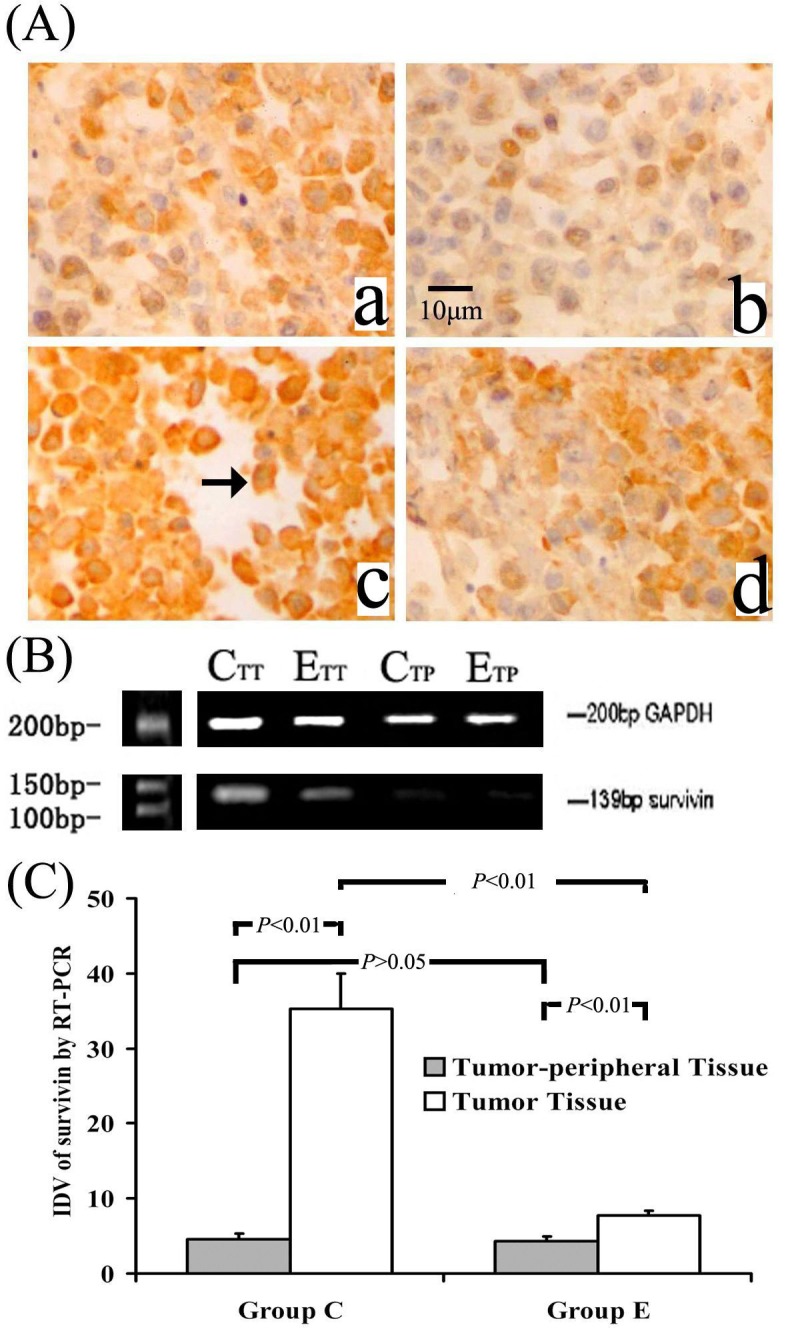
Apoptosis of hepatocarcinoma cells induced by arsenic trioxide (ATO). (**A**) Representative microphotographs of terminal deoxynucleotidyl transferase-mediated dUTP nick-end-labeling (TUNEL) staining performed on liver tissue slices. TUNEL staining of apoptotic VX-2 carcinoma cells showed brown stained nuclei in irregular dotted form of various sizes. The arrowhead indicates representative apoptotic cells. A few apoptotic cells were found in the (a), (b), and (c), (a). Tumor-peripheral tissue in the control group (Group C). (b) Tumor-peripheral tissue in the experimental group (Group E). (c) Tumor tissue in the control group (Group C). (d) Tumor tissue in the experimental group (Group E). Scale bar = 20 μm. (**B**) The bar graphs show the results of apoptotic index in tumor-peripheral and tumor tissues for (Aa), (Ab), (Ac), and (Ad), respectively. The height of each bar represents the mean ± standard deviation. Least-significant difference test was used for multiple comparisons (n = 8).

In the ATO-treated group, significantly more apoptotic cells were observed in tumor tissue than in tumor-peripheral tissue, while in the control group fewer apoptotic cells were found in tumor than tumor-peripheral tissues (Figure 1A). In ATO-treated group, the AI of the tumor tissue was significantly higher than that of the tumor-peripheral tissue (Figure 1 B; *P* < 0.01), and the AI of the tumor tissue in the experimental group was significantly higher than that in the control group (Figure 1 B; *P* < 0.01).

### Down-regulation of survivin expression by ATO in liver tumor

**Figure 2:**
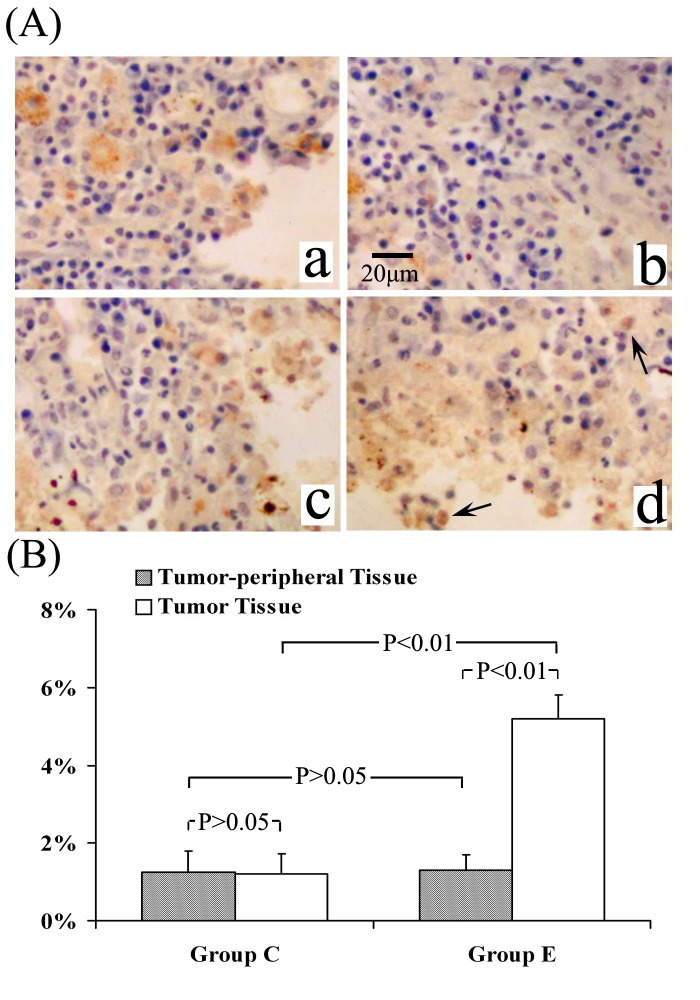
Down-regulation of survivin expression by arsenic trioxide (ATO). (**A**) Representative photomicrographs of immunohistochemical staining detected on liver tissue slices. The arrowhead indicates the representative positive cells. (a) C_TP_ – tumor-peripheral tissue in the control group (Group C); (b) E_TP_ – tumor-peripheral tissue in the experimental group (Group E); (c) C_TT_ – tumor tissue in the control group (Group C); (d) E_TT_ – tumor tissue in the experimental group (Group E). ATO down-regulated the presence of survivin in the tumor tissue of the experimental group (d). Scale bar = 10 μm. (**B**) Expression of survivin mRNA levels was determined by reverse transcription polymerase chain reaction. Survivin transcription in C_TP_, E_TP_, C_TT_, and E_TT_. Glyceraldehyde-3-phosphate dehydrogenase was used as an internal control. (**C**) The integrated density values of survivin mRNA levels were calculated and Tamhane’s T2 test was used for multiple comparisons (n = 8). The height of each bar represents the mean ± standard deviation.

Immunohistochemistry of paraffin-embedded samples revealed a strong expression of survivin protein in tumor tissue in the control group (Figure 2A) and weak expression in the experimental group (Figure 2A). In tumor-peripheral tissue, weak staining was found in both the experimental and control group. In addition, RT-PCR showed that the expression level of survivin mRNA in tumor tissue was significantly lower in experimental than in the control group (Figure 2B and 2C; *P* < 0.01), indicating that the expression of survivin in tumor tissue was down-regulated by ATO. Moreover, the expression level of survivin mRNA was significantly lower in tumor-peripheral tissue than in tumor tissue in both the control and experimental group (Figure 2B and 2C; all *P* < 0.01)

## Discussion

The results showed that in VX-2 carcinoma tissue in the ATO group apoptosis was significantly increased and survivin mRNA and protein expression significantly reduced. This indicates that ATO may induce apoptosis in hepatocellular carcinoma cells, via survivin as the regulatory factor.

Suppression of apoptosis is important for carcinogenesis and tumor growth. Survivin, identified as a new apoptosis inhibitor in the IAP family, plays an important role in gene regulation (11,12). Unlike other members of the IAP family, survivin increases proliferation and decreases apoptosis, which eventually leads to occurrence and growth of tumor (13). It was reported that survivin inhibits apoptosis of tumor cells, and consequently accelerates their proliferative activity (12). Furthermore, the tumor-free 5-year survival rate of patients positive for survivin mRNA was significantly poorer than that of patients negative for survivin mRNA (12). In our study, survivin was much more abundant in VX-2 carcinoma cells than in tumor-peripheral cells. This selective expression may be a self-protecting modification of VX-2 carcinoma cells, which stimulates efficient cell proliferation and tumor growth (14). After treatment with ATO, the expression level of survivin was reduced. Moreover, AI of tumor tissue was higher in the experimental than in the control group. Thus, we hypothesize that survivin might be down-regulated by ATO through a pathway related to apoptosis in VX-2 carcinoma. A detailed mechanism is still unclear and deserves further investigation.

ATO applied through TACE could induce apoptosis, and survivin might play a role in ATO-induced apoptosis in VX-2 carcinoma in the rabbit liver. Moreover, this study suggested that ATO treatment could be a novel and effective therapeutic strategy for liver carcinoma.
